# The activation of PPARγ enhances Treg responses through up-regulating CD36/CPT1-mediated fatty acid oxidation and subsequent N-glycan branching of TβRII/IL-2Rα

**DOI:** 10.1186/s12964-022-00849-9

**Published:** 2022-04-07

**Authors:** Yumeng Miao, Changliu Zhang, Ling Yang, Xi Zeng, Yuxiao Hu, Xinru Xue, Yue Dai, Zhifeng Wei

**Affiliations:** Department of Pharmacology of Chinese Materia Medica, School of Traditional Chinese Pharmacy, China Pharmaceutical University, 24 Tong Jia Xiang, Nanjing, 210009 China

**Keywords:** PPARγ, Treg responses, CD36/CPT1, Fatty acid oxidation, UDP-GlcNAc/glycosylation

## Abstract

**Background:**

Peroxisome proliferator-activated receptor gamma (PPARγ) is an enhancer of Treg responses, but the mechanisms remain elusive. This study aimed to solve this problem in view of cellular metabolism.

**Methods:**

Three recognized PPARγ agonists (synthetic agonist: rosiglitazone; endogenous ligand: 15d-PGJ2; natural product: morin) were used as the tools to activate PPARγ. The fatty acid oxidation (FAO) was evaluated through the detection of fatty acid uptake, oxygen consumption rate, mitochondrial mass, mitochondrial membrane potential and acetyl-CoA level. The involvement of UDP-GlcNAc/N-linked glycosylation axis and the exact role of PPARγ in the action of PPARγ agonists were determined by flow cytometry, Q-PCR, western blotting, a commercial kit for enzyme activity and CRISPR/Cas9-mediated knockout.

**Results:**

Rosiglitazone, 15d-PGJ2 and morin all increased the frequency of CD4^+^Foxp3^+^ Treg cells generated from naïve CD4^+^ T cells, boosted the transcription of Foxp3, IL-10, CTLA4 and TIGIT, and facilitated the function of Treg cells. They significantly promoted FAO in differentiating Treg cells by up-regulating the levels of CD36 and CPT1 but not other enzymes involved in FAO such as ACADL, ACADM, HADHA or HADHB, and siCD36 or siCPT1 dampened PPARγ agonists-promoted Treg responses. Moreover, PPARγ agonists enhanced UDP-GlcNAc biosynthesis and subsequent N-linked glycosylation, but did not affect the expressions of N-glycan branching enzymes Mgat1, 2, 4 and 5. Notably, the enzyme activity of phosphofructokinase (PFK) was inhibited by PPARγ agonists and the effect was limited by siCD36 or siCPT1, implying PFK to be a link between PPARγ agonists-promoted FAO and UDP-GlcNAc biosynthesis aside from acetyl-CoA. Furthermore, PPARγ agonists facilitated the cell surface abundance of TβRII and IL-2Rα via N-linked glycosylation, thereby activating TGF-β/Smads and IL-2/STAT5 signaling, and the connection between N-linked glycosylation and Treg responses was revealed by tunicamycin. However, the increased surface abundance of CD36 was demonstrated to be mainly owing to PPARγ agonists-up-regulated overall expression. Finally, PPARγ antagonist GW9662 or CRISPR/Cas9-mediated knockout of PPARγ constrained the effects of rosiglitazone, 15d-PGJ2 and morin, confirming the exact role of PPARγ.

**Conclusions:**

The activation of PPARγ enhances Treg responses through up-regulating CD36/CPT1-mediated fatty acid oxidation and subsequent N-glycan branching of TβRII/IL-2Rα, which is beneficial for inflammatory and autoimmune diseases.

**Graphical abstract:**

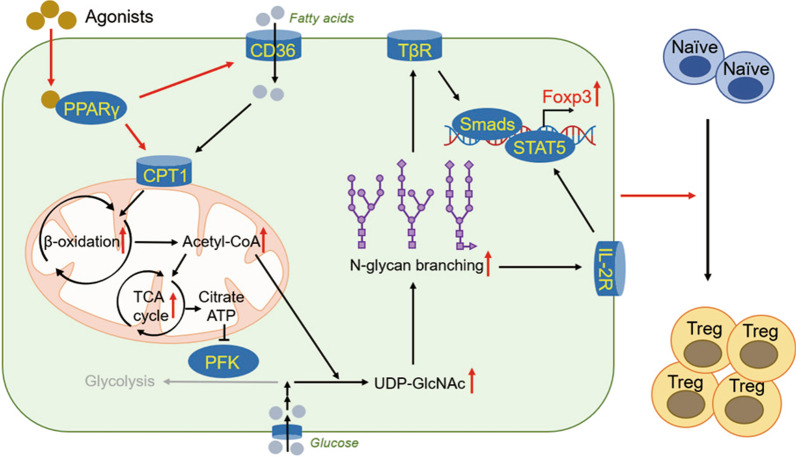

**Video Abstract**

**Supplementary Information:**

The online version contains supplementary material available at 10.1186/s12964-022-00849-9.

## Background

Regulatory T (Treg) cells are a suppressive subset of CD4^+^ T cells that polarize under the control of forkhead box protein 3 (Foxp3). By preventing the dysregulated activation of cells within both the innate and adaptive arms of the immune system, and limiting their ability to release inflammatory cytokines, Treg cells play a central role in maintenance of immune homeostasis and ensure that the responses of immune system to foreign- or self-antigens remain adequately balanced [1, 2]. As deficits in Treg number and function lead to immune dysfunctions, loss of tolerance and autoimmune phenomena, the therapy of Treg enhancing has emerged as a focus aimed at curing or controlling inflammation and autoimmune diseases, such as inflammatory bowel disease (IBD), rheumatoid arthritis (RA), multiple sclerosis (MS) and so on [3, 4].

With the respect to determine the molecular targets for regulating Treg cells, intensive research endeavors have been invested and an emphasis can be given to peroxisome proliferator-activated receptor gamma (PPARγ), a transcription factor long been considered as the target protein of thiazolidinediones [5]. It has been reported that PPARγ agonist ciglitazone can promote Foxp3 gene transcription, induce Treg cell generation, and enhance the anti-proliferation ability of Treg cells [6]. In the absence of TGF-β, the key stimulator for Treg differentiation, pioglitazone is still able to elevating the frequency of CD4^+^CD25^+^Foxp3^+^ Treg cells, of which the effect can be blocked by PPARγ antagonist GW9662 [7]. Moreover, PGJ2 weakens the inhibition of advanced glycation end products (AGEs) on Treg function [8], and adoptive transfer of PPARγ-deficient Treg cells has a significantly weaker effect than wild-type cells on the prevention and treatment of colitis in SCID mice [9]. Based on these, a clear link between PPARγ and Treg cells can be obtained. However, the in-depth mechanisms of PPARγ as a promising molecular target for specific intervention in Treg responses are still in doubt and need more investigation.

Only recently, were the intrinsic metabolic programming established as a key component for the differentiation and function of immune cells. Unlike T helper (Th) CD4^+^ T cells, which are strongly biased toward glycolysis, glutaminolysis or fatty acid synthesis, Treg cells preferentially use fatty acid oxidation (FAO) for their energy supply, and the enhanced FAO has been reported to result in stronger Treg responses [10–14]. Of note, PPARγ is a critical regulator of cellular metabolism. By interfering with FAO, PPARγ participates in M2 macrophage polarization and possesses protective effects on obesity as well as pulmonary hypertension [15–17]. Therefore, in this study, we determined the effect of PPARγ on Treg responses by using three recognized PPARγ agonists (synthetic agonist: rosiglitazone; endogenous ligand: 15d-PGJ2; natural product: morin), and further explored the underlying mechanisms from the perspective of fatty acid oxidation.

## Materials and methods

### Animals

C57BL/6 mice, weighing 18–22 g, were purchased from the Comparative Medicine Centre of Yangzhou University (Yangzhou, China). The animal treatments were conducted with the approval of the Animal Ethics Committee of China Pharmaceutical University and complied with the National Institute of Health guidelines on the ethical use of animals. All animals were housed under a 12 h light/dark cycle (21 ± 2 °C) and allowed ad libitum access to a diet of standard laboratory chow and water.

### Chemicals and reagents

Rosiglitazone and GW9662 were purchased from TargetMol (Shanghai, China). 15d-PGJ2 was purchased from Sigma-Aldrich (St. Louis, USA). Morin (3, 5, 7, 2′, 4′-pentahydroxyflavone, a flavonoid exists in *Morus alba* L. (Moraceae), *Otostegia persica* (Lamiaceae), etc.; purity > 98%) was purchased from Nanjing JingZhu Biological Technology Co., Ltd. (Nanjing, China). Mouse CD4^+^CD62L^+^ T-cell isolation kit was purchased from Miltenyi Biotech (Cologne, Germany), and rhTGF-β1 was purchased from R&D Systems (Minneapolis, USA). Purified anti-mouse CD3/CD28 mAbs, PE-anti-Foxp3, and fixation/permeabilization concentrate and diluent reagent were purchased from eBioscience (San Diego, USA). FITC-anti-CD4 and PE-anti-CD36 was purchased from BioLegend (San Diego, USA). Antibodies against TβRII, CPT1 and PPARγ were purchased from Proteintech (Wuhan, China). Antibodies against Smad3, STAT5 and pSTAT5 were purchased from Wanleibio (Shenyang, China). Antibody against β-actin was purchased from Bioworld Technology, Inc. (Nanjing, China). Antibody against CD36 and pSmad3, MitoTracker Green and mitochondrial membrane potential assay kit with JC-1 and tunicamycin were purchased from Beyotime (Nantong, China). Actinomycin D (Act-D) was purchased from MedChemexpress (Monmouth Junction, USA). BODIPY-C16 and lipofectamine 2000 was purchased from Invitrogen (Carlsbad, USA). FITC-L-PHA was purchased from Vectorlabs (Burlingame, USA). TRIzol, HiScript^™^ reverse transcriptase system and AceQ^™^ qPCR SYBR^®^ Green Master Mix were purchased from Vazyme (Nanjing, China).

### Cell culture and differentiation

Naïve CD4^+^ T cells isolated from mesenteric lymph nodes (MLNs) of mice were purified with magnetic beads according to the manufacturer’s instructions for the CD4^+^CD62L^+^ T-cell isolation kit. The cells were maintained in RPMI 1640 (Gibco, Carlsbad, USA) contained 10% fetal bovine serum (FBS) at 37 °C under a humidified 5% (v/v) CO_2_ atmosphere.

For Treg cell differentiation, naïve CD4^+^ T cells were treated with plate-bound anti-CD3 (1 μg/mL), anti-CD28 (1 μg/mL) and rhTGF-β1 (5 ng/mL) for 72 h. At the end of the differentiation, the cells were harvested and stained with FITC-anti-CD4 for 30 min at 4 °C, followed by fixation and permeabilization for 5 h. Then, the cells were stained with PE-anti-Foxp3 for another 1 h, washed and resuspended for detection by flow cytometry.

### Cell viability assay

The cells were seeded into a 96-well plate at a density of 1 × 10^6^ cells/mL and treated with rosiglitazone (1, 3, 10, 30, 100 μM), 15d-PGJ2 (1, 3, 10, 30, 100 μM) or morin (1, 3, 10, 30, 100 μM) for 72 h.

For MTT assay, 20 μL of MTT (Beyotime, Nantong, China) solution (5 mg/mL dissolved in PBS) was added into each well 4 h before the end of incubation. Then, the supernatant was discarded, and 150 μL of DMSO was added to dissolve the crystals. The optical absorbance value was measured at 570 nm.

For CCK-8 assay, 10 μL of CCK-8 (Beyotime, Nantong, China) was added to each well 4 h before the end of incubation, and the optical absorbance value was measured at 450 nm.

### Western blotting assay

The cell lysates were prepared by using NP-40 buffer (Beyotime, Nantong, China) which contained 1 mM PMSF (Beyotime, Nantong, China), and were centrifuged at 12,000 rpm for 10 min. The supernatants were collected and mixed with the loading buffer (Beyotime, Nantong, China). After being boiled for 10 min, all samples were separated by 10% SDS-PAGE, and the gels were transferred onto the pre-activated polyvinylidene fluoride (PVDF) membranes (Millipore, Billerica, USA). Then, the membranes were blocked with 9% nonfat milk for 2 h at room temperature, and probed with primary antibodies overnight at 4 °C, followed by incubation with horseradish peroxide (HRP)-conjugated secondary antibodies for 2 h at room temperature. The blots were finally visualized by using enhanced chemiluminescent (ECL) reagent (Vazyme, Nanjing, China).

### Q-PCR assay

The total RNAs of cells were extracted by TRIzol reagent. Then, the HiScript^™^ reverse transcriptase system was used to reverse transcribe the RNAs into cDNAs. Finally, Q-PCR assay of target genes was performed using AceQ^™^ qPCR SYBR^®^ Green Master Mix and gene-specific primers on the Bio-Rad CFX Connect real-time PCR system (Bio-Rad, Hercules, USA). The mRNA expression levels were normalized to β-actin. In addition, the details of the gene-specific primers (Sangon Biotech, Shanghai, China) were listed in Table 1.

**Table 1:** Primers used in Q-PCR

Primers		Sequence (5′–3′)
IL-10 (mouse)	Forward	TTCTTTCAAACAAAGGACCAGC
	Reverse	GCAACCCAAGTAACCCTTAAAG
CTLA4 (mouse)	Forward	CGCAGATTTATGTCATTGATCCAGAACC
	Reverse	CAAAGAAACAGCAGTGACCAGGAAAC
TIGIT (mouse)	Forward	CAGCAGGCACGATAGATACAAAGAGG
	Reverse	CAGAGGAGAAGTGACACTGTAAGATGAC
Foxp3 (mouse)	Forward	TTTCACCTATGCCACCCTTATC
	Reverse	CATGCGAGTAAACCAATGGTAG
CD36 (mouse)	Forward	CTTTGAAAGAACTCTTGTGGGG
	Reverse	GTCTGTGCCATTAATCATGTCG
CPT1 (mouse)	Forward	CTACATCACCCCAACCCATATT
	Reverse	GATCCCAGAAGACGAATAGGTT
ACADL (mouse)	Forward	AAACAGTTGCACACATACAGAC
	Reverse	ATTCAGATGCCCAGTATTTTGC
ACADM (mouse)	Forward	TCAGAGTGCCTAAGGAAAATGT
	Reverse	CGACTGTAGGTCTGGTTCTATC
HADHA (mouse)	Forward	GCAGACGAAGTGGGTGTGGATG
	Reverse	CTTGCGACCTAAGAAGCCCTTGG
HADHB (mouse)	Forward	GCTGCCTTTGCTGTTTCTCGAATG
	Reverse	GTCCTTCATCCTGTGCCTTCTTGG
Mgat1 (mouse)	Forward	CAGCTACAGGTGGAGAAAGTAA
	Reverse	GAAGCTGTCTCTGCTAGTGTAC
Mgat2 (mouse)	Forward	AGCAATGGGCGACAAAGGAAGAG
	Reverse	ATTCGTTTGAGACCCTGCGGATG
Mgat4 (mouse)	Forward	GATAGAGTCAGATTTCGCTCCA
	Reverse	GAAATTTCTGGAACACCGAACA
Mgat5 (mouse)	Forward	CGTCTGGACTGTGGATCTCAATAACC
	Reverse	ATGGCATATACGGCTCAATCTTCTGG
GFPT (mouse)	Forward	TAAGGCACTGGATGAAGAAGTT
	Reverse	CGGGTATGAGCTATTCCAAGAT
PFK (mouse)	Forward	GATTGGGAGGAAAATATGTGCC
	Reverse	GCTTATTTTGCATGTCGATTGC
TβRII (mouse)	Forward	GACCTCAAGAGCTCTAACATCC
	Reverse	GTCATCCACAGACAGAGTAGG
IL-2Rα (mouse)	Forward	AGAGGTTTCCGAAGACTAAAGG
	Reverse	TTCAAGTTGAGCTGTAACTTGC

### Measurement of fatty acid uptake

The naïve CD4^+^ T cells were prepared and treated with anti-CD3/CD28 in the presence or absence of TGF-β (5 ng/mL), rosiglitazone (10, 30 μM), 15d-PGJ2 (3, 10 μM) as well as morin (10, 30 μM) for 48 h. Then, the cells were collected, washed and resuspended in PBS contained BODIPY-C16 (100 nM) for a 30 min-incubation at 37 °C. At the end of the incubation, the cells were washed and the uptake of BODIPY-C16 was detected by flow cytometry.

### Measurement of oxygen consumption rate (OCR)

The OCR was measured with a Seahorse XF96 bioanalyzer using the XF palmitate-BSA FAO substrate (Seahorse Bioscience, North Billerica, USA) and Mito Stress Test Kit (Seahorse Bioscience, North Billerica, USA) according to the manufacturer’s instructions. The OCR for oxidation of palmitate-BSA was measured in cells treated with palmitate-BSA (30 μL/well), oligomycin (1.5 μM), carbonyl cyanide 4-(trifluoromethoxy) phenylhydrazone (FCCP; 2 μM), and rotenone/antimycin A (0.5 μM).

### Measurement of mitochondrial mass

The naïve CD4^+^ T cells were prepared and treated with anti-CD3/CD28 in the presence or absence of TGF-β (5 ng/mL), rosiglitazone (10, 30 μM), 15d-PGJ2 (3, 10 μM) as well as morin (10, 30 μM) for 48 h. Then, the cells were incubated with MitoTracker Green (100 nM) at 37 °C for 30 min. After staining, the cells were collected and resuspended in fresh media. The mitochondrial mass was observed with a fluorescence microscope (Olympus IX51).

### Measurement of mitochondrial membrane potential

The naïve CD4^+^ T cells were prepared and treated with anti-CD3/CD28 in the presence or absence of TGF-β (5 ng/mL), rosiglitazone (10, 30 μM), 15d-PGJ2 (3, 10 μM) as well as morin (10, 30 μM) for 48 h. The change of mitochondrial membrane potential was measured with a JC-1 assay kit according to manufacturer’s instruction. Briefly, JC-1 stock solution (200×) was diluted to working solution (1×) and added to the cells for a 20 min-incubation at 37 °C. Then, the cells were washed with washing buffer (1×), and the fluorescence intensity was measured by flow cytometry. The ratio of JC-1 red/green fluorescence was calculated using mean fluorescence intensity of each channel.

### Measurement of uridine diphosphate-*N*-acetylglucosamine (UDP-GlcNAc)

UDP-GlcNAc was measured as previously described [18]. Briefly, the cells were collected, washed, and lysed by addition of 200 μL of chloroform/water (1:1). Then, the samples were vortexed for 2 min and centrifuged at 15,000 × g for 20 min at 4 °C. The aqueous phase was transferred to a fresh tube and 10 μL of HCl (1 N) was added to hydrolyze UDP-GlcNAc to GlcNAc. After heating for 20 min at 80 °C, the sample was neutralized with 10 μL KOH (1 N). Next, 50 μL of potassium tetraborate (200 mM) was added, and the samples were heated at 80 °C for 25 min and then cooled on ice for 5 min. 150 μL of Ehrlich's reagent (diluted 1:2 in acetic acid) was added to the samples followed by a 20 min-incubation at 37 °C. Finally, the samples were centrifuged at 15,000 × g for 20 min, and the absorbance was measured at 595 nm.

### Measurement of N-linked glycosylation

For the measurement of N-linked glycosylation, the cells were collected, washed, and incubated with PBS contained FITC-L-PHA (5 μg/mL) for 30 min at 4 °C. Then, the cells were washed again for flow cytometry.

### Measurement of phosphofructokinase (PFK) and glutamine-fructose-6-phosphate transaminase (GFPT) enzymatic activity

The PFK enzymatic activity was measured using a commercial kit (Jiancheng Bioengineering Institute, Nanjing, China) according to the manufacturer's instructions.

The GFPT enzymatic activity was measured as previously described [19] with the following modifications. Cells were sonicated in extraction buffer (60 mM KH2PO4, pH7.8, 50 mM KCl, 1 mM EDTA, 1 mM DTT) and the lysate was collected. Enzymatic reactions were performed in reaction buffer (60 mM KH2PO4, pH7.8, 50 mM KCl, 1 mM EDTA, 1 mM DTT, 15 mM fructose-6-P, 15 mM l-glutamine) at 37 °C for 1 h and terminated by heating up at 95 °C for 2 min. In negative reaction, all the proteins were denatured right away by heating up at 95 °C for 2 min and therefore, the original level of glutamate was measured for background deduction. After centrifugation, the supernatant was collected and the byproduct from the reaction, glutamate, was quantified using a glutamate assay kit (Solaibao Biotechnology Co., Ltd., Beijing, China) following the manufacturer’s instructions.

### Cell transfection

For knockdown experiments, cells were transfected with mouse siCD36 (sense: 5′-CUGAGUAGGUUUUUCUCUU-3′; anti-sense: 5′-AAGAGAAAAACCUACUCAG-3′), siCPT1 (sense: 5′-GGUUCAAGCUGUUCAAGAUAG-3′; anti-sense: 5′-AUCUUGAACAGCUUGAACCUC-3′) or siPFK (sense: 5′-CAAGAUGUUUGCAAUCUAUGA-3′; anti-sense: 5′-AUAGAUUGCAAACAUCUUGUG-3′) synthesized by RiboBio Co. (Guangzhou, China). For knockout experiments, cells were transfected with PPARγ CRISPR/Cas9 KO plasmid designed and constructed by Genomeditech (Shanghai, China). Transfection was performed by using Lipofectamine 2000 according to the manufacturer’s instructions.

### Chromatin-immunoprecipitation (ChIP) assay

The ChIP assay was performed by using a commercial kit (Beyotime, Nantong, China) according to the manufacturer’s instruction. Briefly, the cells were harvested and treated with 1% formaldehyde for 10 min at 37 °C for crosslinking, and the reaction was quenched by adding glycine. The cells were washed with PBS containing 1 mM PMSF and resuspended in SDS lysis buffer (including 1 mM PMSF) for subsequent sonication (amplitude, 40 W; process time, 6 min; ON time, 4.5 s; OFF time, 9 s). Immunoprecipitation was further carried out by adding antibody against PPARγ and protein A + G Agarose/Salmon Sperm. Moreover, the protein-DNA complexes were de-crosslinked at 65 °C for 4 h, followed by proteinase K treatment so as to degrade the protein. The acquired DNAs were purified by using the DNA purification kit (Beyotime, Nantong, China), and the enrichment was detected by Q-PCR. The primers used were listed as follows: CD36 promoter (forward), 5′-CAGGCTTTGTTGGGACAGAC-3′; (reverse), 5′-GCTAATTTGTGGTTGGTTGCCA-3′; CPT1 promoter (forward), 5′-CTGGAGAGGAATGGGACAAC-3′; (reverse), 5′-ATTGGGGTGGAGAAAACAGA-3′.

### Statistical analysis

All the data were analyzed by SPSS software (SPSS, Chicago, USA) and presented as the mean ± S.E.M. Independent-Samples T test was performed to compare the mean differences between two groups. One-way ANOVA followed by the LSD test was conducted to compare the mean differences between multiple groups, and in cases where the latter condition was violated, non-parametric Games-Howell post hoc test was used. A value of *p* less than 0.05 (*p* < 0.05) was accepted as a significant difference.

## Results

### PPARγ agonists promote the generation and function of Treg cells

**Fig. 1:**
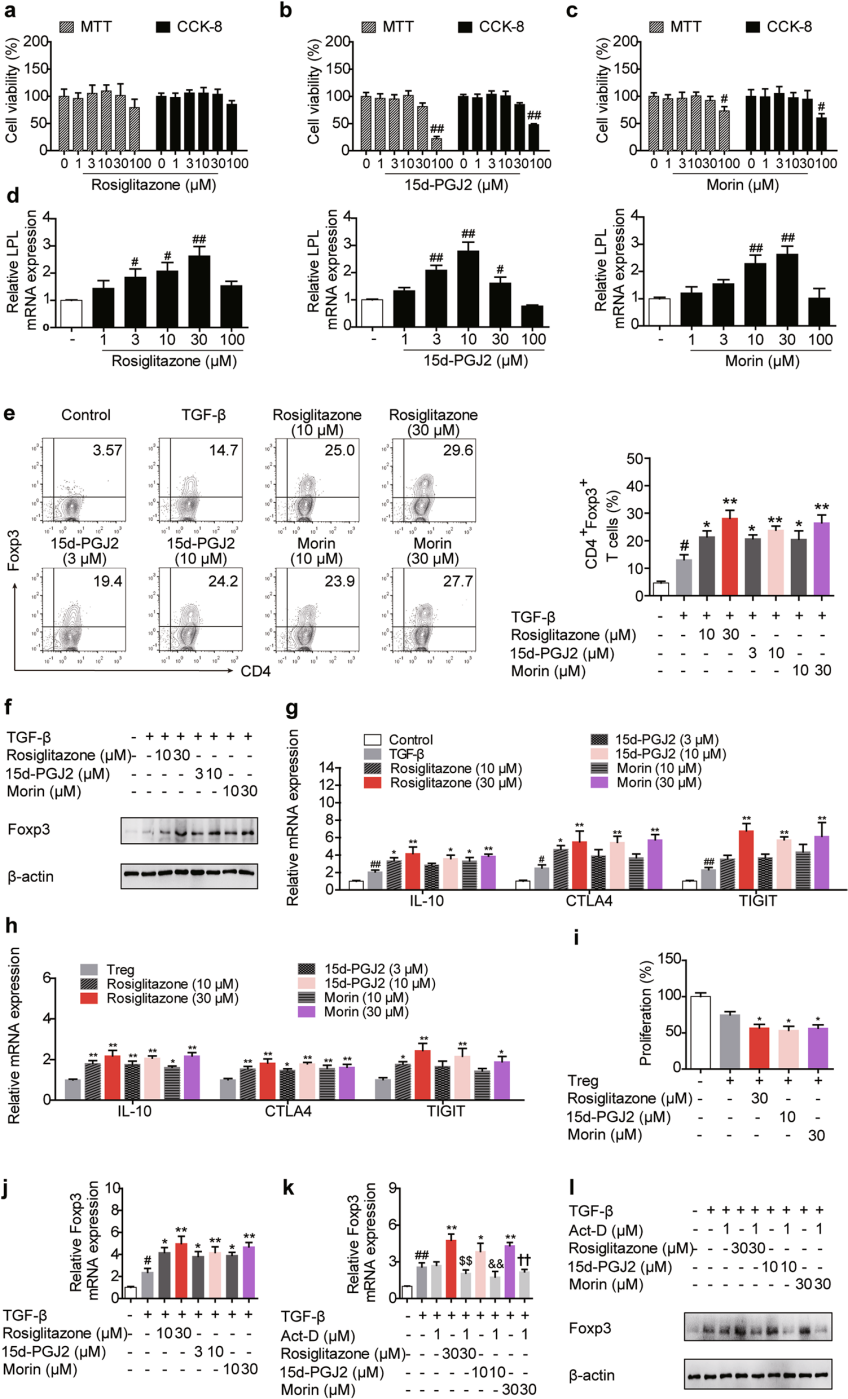
PPARγ agonists promote the generation and function of Treg cells. **a**–**d** The cells isolated from mesenteric lymph nodes of mice were cultured with rosiglitazone (0, 1, 3, 10, 30, 100 μM), 15d-PGJ2 (0, 1, 3, 10, 30, 100 μM) and morin (0, 1, 3, 10, 30, 100 μM). Cell viability was analyzed by MTT and CCK-8 assay after 72 h, and the mRNA expression of LPL was measured by Q-PCR after 24 h. **e**–**g** The naïve CD4^+^ T cells were prepared and treated with anti-CD3/CD28 in the presence or absence of TGF-β (5 ng/mL), rosiglitazone (10, 30 μM), 15d-PGJ2 (3, 10 μM) as well as morin (10, 30 μM) for 72 h. The frequency of CD4^+^Foxp3^+^ T cells was examined by flow cytometry (**e**), the protein level of Foxp3 was detected by western blotting (**f**), and the mRNA expression levels of IL-10, CTLA4 and TIGIT were measured by Q-PCR (**g**). **h** The naïve CD4^+^ T cells were stimulated under Treg differentiation conditions for 72 h, and then treated with anti-CD3/CD28 in the presence or absence of rosiglitazone (30 μM), 15d-PGJ2 (10 μM) as well as morin (30 μM) for another 72 h. The mRNA expression levels of IL-10, CTLA4 and TIGIT were measured by Q-PCR. **i** The naïve CD4^+^ T cells were stimulated under Treg differentiation conditions for 72 h, and then co-cultured with CFSE-labeled CD4^+^ T cells under the stimulation of anti-CD3/CD28 in the presence or absence of rosiglitazone (30 μM), 15d-PGJ2 (10 μM) as well as morin (30 μM) for another 72 h. The suppressive activity of Treg cells were examined by flow cytometry. **j** The naïve CD4^+^ T cells were prepared and treated with anti-CD3/CD28 in the presence or absence of TGF-β (5 ng/mL), rosiglitazone (10, 30 μM), 15d-PGJ2 (3, 10 μM) as well as morin (10, 30 μM) for 72 h. The mRNA expression level of Foxp3 was measured by Q-PCR. **k**, **l** The naïve CD4^+^ T cells were prepared and treated with anti-CD3/CD28 in the presence or absence of TGF-β (5 ng/mL), actinomycin D (Act-D; 1 μM), rosiglitazone (30 μM), 15d-PGJ2 (10 μM) as well as morin (30 μM) for 72 h. The mRNA expression level of Foxp3 was measured by Q-PCR (**k**), and the protein level of Foxp3 was detected by western blotting (**l**). Data were presented as the means ± S.E.M. of three independent experiments (n = 3). ^#^*P* < 0.05, ^##^*P* < 0.01 versus control group; *P < 0.05, **P < 0.01 versus TGF-β group (model group); ^$$^*P* < 0.01 versus rosiglitazone group; ^&&^*P* < 0.01 versus 15d-PGJ2 group; ^††^*P* < 0.01 versus morin group

To evaluate the effects of PPARγ on Treg responses, the cytotoxicity of the tool drugs, three PPARγ agonists including rosiglitazone, 15d-PGJ2 and morin, was firstly examined. The results revealed that at the concentration below 100 μM, rosiglitazone and morin did not affect the viability of lymphocytes, while the safe concentration was lower in the case of 15d-PGJ2 as slight cytotoxicity exhibited when the concentration reached 30 μM (Fig. 1a–c). By measuring the mRNA expression of PPARγ target gene LPL, it was found that rosiglitazone (30 μM), 15d-PGJ2 (10 μM) and morin (30 μM) acted with a similar intensity in lymphocytes (Fig. 1d). As shown in Fig. 1e, f, after incubation with rosiglitazone (10, 30 μM), 15d-PGJ2 (3, 10 μM) and morin (10, 30 μM), more CD4^+^Foxp3^+^ T cells were generated compared with untreated cells, and the protein level of Treg-specific transcription factor Foxp3 was distinctively increased by them, indicating that PPARγ agonists-stimulated T cells were more prone to differentiate towards Treg cells. In addition, rosiglitazone (30 μM), 15d-PGJ2 (10 μM) and morin (30 μM) favored the mRNA expressions of the function-related factors such as IL-10, CTLA4 and TIGIT in differentiating or differentiated Treg cells, and enhanced the suppressive activity as well (Fig. 1g–i). However, as revealed by Edu and Annexin V-FITC/PI staining, PPARγ agonists exerted only slight effects on the proliferation and apoptosis of cells during Treg differentiation (Additional file 1: Fig. S1).

Considering that Foxp3 is the master regulator for differentiation and function of Treg cells [20], the effect of PPARγ agonists on it was explored for detail. It was found that the mRNA expression of Foxp3 was boosted by rosiglitazone (10, 30 μM), 15d-PGJ2 (3, 10 μM) and morin (10, 30 μM) (Fig. 1j), while the RNA synthesis inhibitor Act-D could restrict the increasing effects of PPARγ agonists on both the mRNA and protein levels of Foxp3 (Fig. 1k, l), implying a regulatory role PPARγ played at the transcriptional level. These findings suggest that PPARγ agonists can facilitate the generation and function of Treg cells.

### CD36/CPT1-mediated FAO participates in PPARγ agonists-induced Treg differentiation

**Fig. 2:**
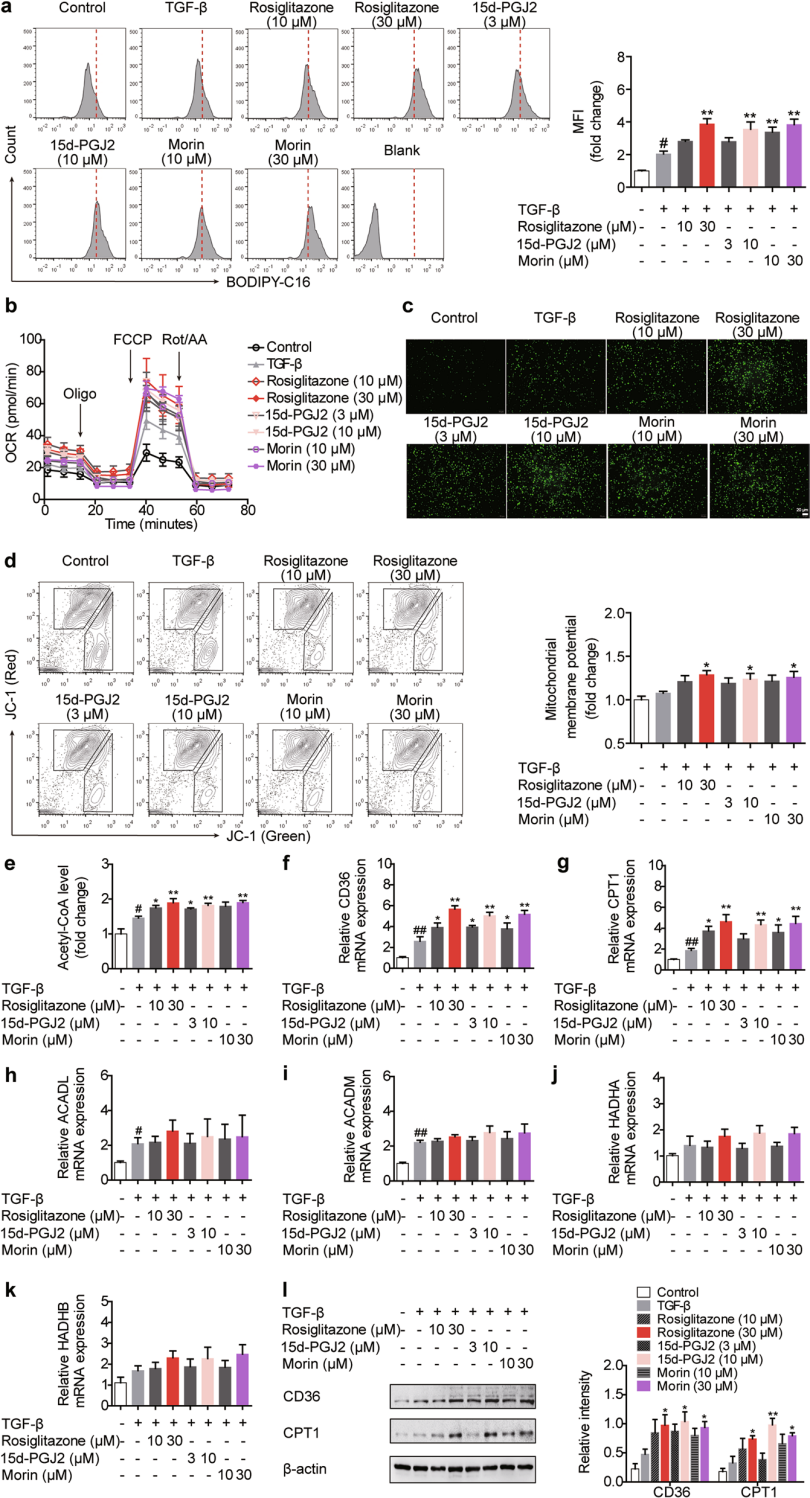
PPARγ agonists induce CD36/CPT1-mediated FAO during Treg differentiation. The naïve CD4^+^ T cells were prepared and treated with anti-CD3/CD28 in the presence or absence of TGF-β, rosiglitazone (10, 30 μM), 15d-PGJ2 (3, 10 μM) as well as morin (10, 30 μM) for 48 h. **a** The fatty acid uptake was examined by flow cytometry using BODIPY-C16. **b** The OCR was monitored by Seahorse XFe 96 analyzer. **c** The mitochondrial mass was observed with a fluorescence microscope using MitoTracker Green. **d** The mitochondrial membrane potential was analyzed by flow cytometry using a JC-1 assay kit. **e** The level of acetyl-CoA was determined using a commercial acetyl-CoA assay kit. **f**–**k** The mRNA expression levels of CD36, CPT1, ACADL, ACADM, HADHA as well as HADHB were measured by Q-PCR. **l** The protein levels of CD36 and CPT1 were detected by western blotting. Data were presented as the means ± S.E.M. of three independent experiments (n = 3). ^#^*P* < 0.05, ^##^*P* < 0.01 versus control group; *P < 0.05, **P < 0.01 versus TGF-β group (model group)

The β-oxidation of exogenous fatty acid is a critical energy source for Treg cells. This multi-step process begins with the uptake of exogenous fatty acid into the cytosol and allows for the mitochondrial conversion into acetyl-coenzyme A (acetyl-CoA) [21]. The results in Fig. 2a–e revealed a connection between PPARγ and FAO, since rosiglitazone (10, 30 μM), 15d-PGJ2 (3, 10 μM) and morin (10, 30 μM) significantly promoted the uptake of BODIPY-C16 (a kind of fluorescently labeled palmitate applied as the indicator for fatty acid uptake), up-regulated palmitate-based OCR, increased the mitochondrial mass, and up-regulated the mitochondrial membrane potential as well as the content of acetyl-CoA. The expressions of CD36 and CPT1, two enzymes involved in the process of FAO, were elevated by PPARγ agonists, further consolidating the connection, although the expression levels of ACADL, ACADM, HADHA or HADHB were not distinctively affected (Fig. 2f–l).

**Fig. 3:**
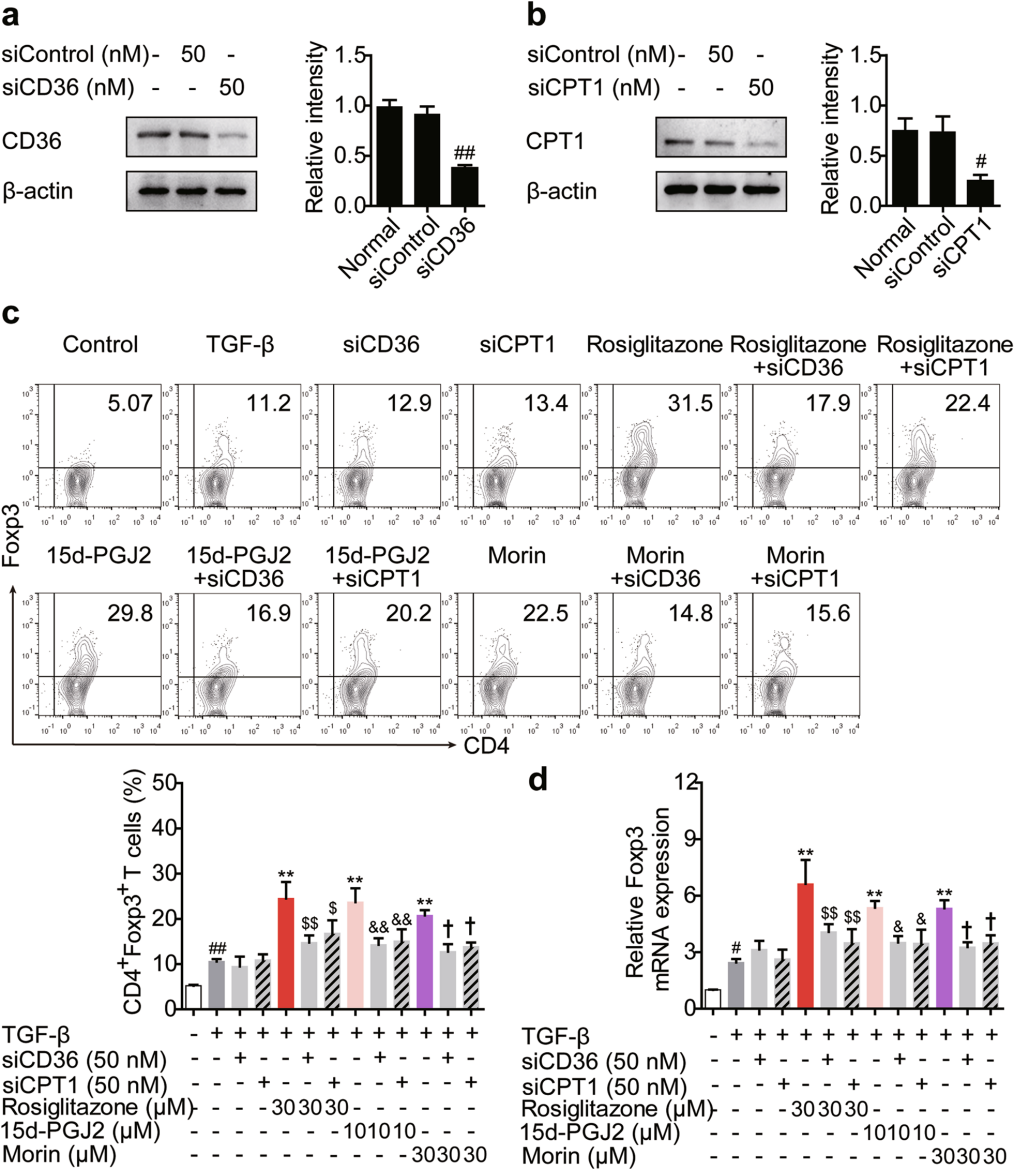
The Treg-promoting effect of PPARγ agonists is dampened by knocking down CD36/CPT1. **a**, **b** The naïve CD4^+^ T cells were transfected with siCD36 or siCPT1, and the protein levels of CD36 (**a**) and CPT1 (**b**) were detected by western blotting. **c**, **d** The naïve CD4^+^ T cells were transfected with siCD36 or siCPT1, and treated with anti-CD3/CD28 in the presence or absence of TGF-β (5 ng/mL), rosiglitazone (30 μM), 15d-PGJ2 (10 μM) as well as morin (30 μM) for 72 h. The frequency of CD4^+^Foxp3^+^ T cells was examined by flow cytometry (**c**) and the mRNA expression level of Foxp3 was measured by Q-PCR (**d**). Data were presented as the means ± S.E.M. of three independent experiments (n = 3). ^#^*P* < 0.05, ^##^*P* < 0.01 versus control group; **P < 0.01 versus TGF-β group (model group); ^$^*P* < 0.05, ^$$^*P* < 0.01 versus rosiglitazone group; ^&^*P* < 0.05, ^&&^*P* < 0.01 versus 15d-PGJ2 group; ^†^*P* < 0.05 versus morin group

In addition, to determine whether the CD36/CPT1-mediated FAO participated in PPARγ agonists-induced Treg differentiation, small interfering RNA was used. As expected, by knocking down CD36 or CPT1, the promoting effects of PPARγ agonists on the frequency of CD4^+^Foxp3^+^ T cells and the mRNA expression of Foxp3 were reduced (Fig. 3). Therefore, PPARγ agonists induce Treg differentiation mainly through enhancing CD36/CPT1-mediated FAO.

### PPARγ agonists increase UDP-GlcNAc level and N-glycan branching during Treg differentiation

**Fig. 4:**
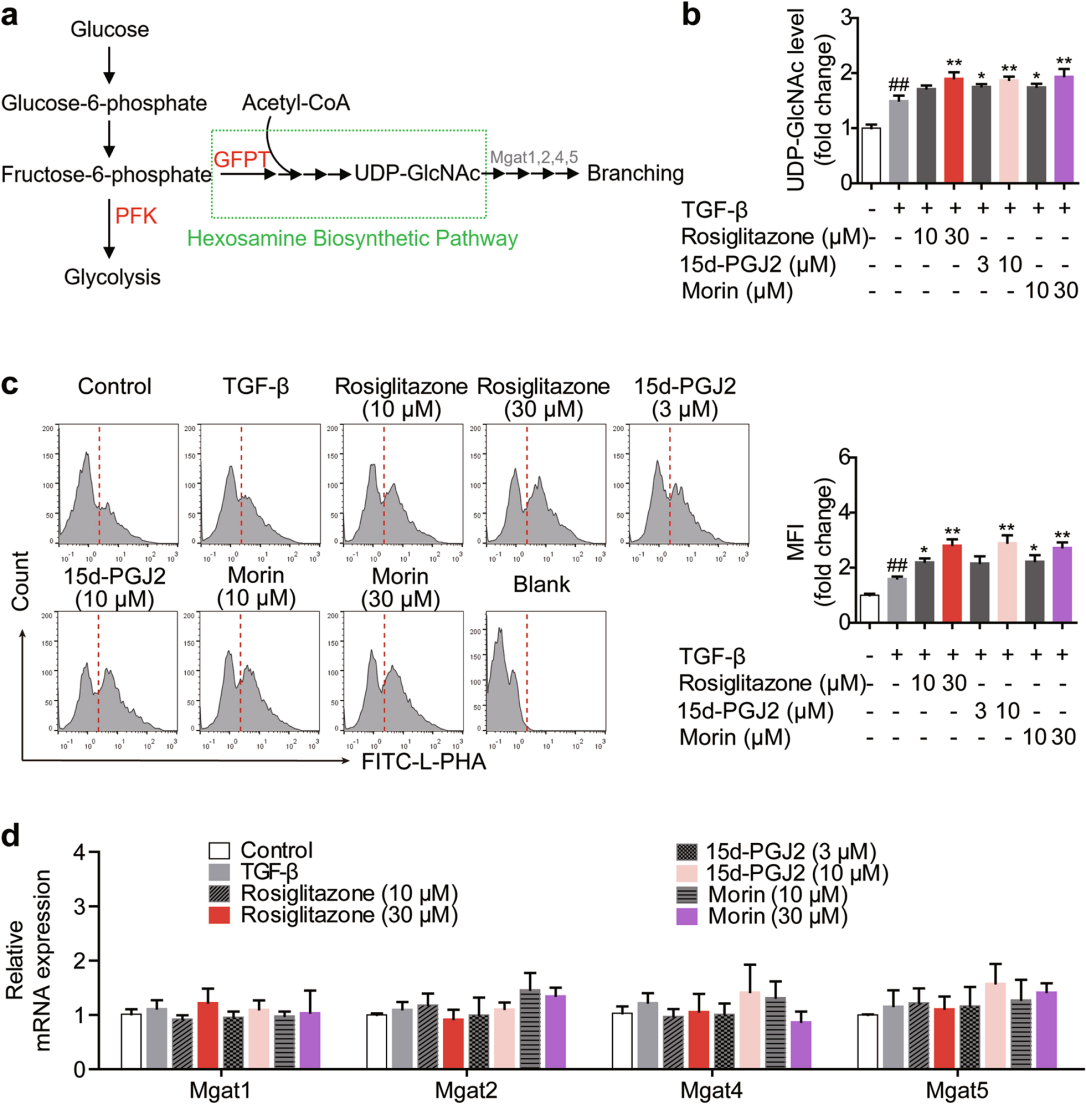
PPARγ agonists increase the level of UDP-GlcNAc and subsequent N-glycan branching during Treg differentiation. **a** The process of hexosamine biosynthesis pathway and the N-glycan biosynthesis was shown. **b**–**d** The naïve CD4^+^ T cells were prepared and treated with anti-CD3/CD28 in the presence or absence of TGF-β (5 ng/mL), rosiglitazone (10, 30 μM), 15d-PGJ2 (3, 10 μM) as well as morin (10, 30 μM) for 48 h. The intracellular level of UDP-GlcNAc was detected (**b**), the level of N-linked glycosylation was examined by flow cytometry using FITC-L-PHA (**c**), and the mRNA expression levels of Mgat1, Mgat2, Mgat4 and Mgat5 (**d**) were measured by Q-PCR. ^##^*P* < 0.01 versus control group; *P < 0.05, **P < 0.01 versus TGF-β group (model group)

It is worth noting that acetyl-CoA derived from FAO is the building block of UDP-GlcNAc, the biosynthesis of which is also known as the hexosamine biosynthetic pathway (HBP) [22]. Moreover, UDP-GlcNAc is the sugar-nucleotide donor substrate required by the N-glycan branching enzymes Mgat1, 2, 4 and 5 during the process of N-linked glycosylation (Fig. 4a), and the UDP-GlcNAc/glycosylation axis has been reported to play an essential role in Treg differentiation [23, 24]. Thus, attention was focused on UDP-GlcNAc and N-linked glycosylation in order to gain insight into the mechanisms of PPARγ agonists subsequent to FAO.

As shown in Fig. 4b, rosiglitazone (10, 30 μM), 15d-PGJ2 (3, 10 μM) and morin (10, 30 μM) obviously up-regulated the level of UDP-GlcNAc in differentiating Treg cells, and the enhanced mean fluorescence intensity (MFI) of FITC-L-PHA could be seen as well (Fig. 4c), indicating the increased N-glycan branching caused by them. However, the possibility that PPARγ agonists exerted a direct effect on N-glycan branching could be ruled out, as rosiglitazone (10, 30 μM), 15d-PGJ2 (3, 10 μM) and morin (10, 30 μM) barely altered the mRNA expressions of Mgat1, Mgat2, Mgat4 and Mgat5 (Fig. 4d), confirming that the effect of PPARγ agonists on N-glycan branching was attributed to the increased supply of UDP-GlcNAc. These results suggest that PPARγ agonists enhance the UDP-GlcNAc/glycosylation axis during Treg differentiation.

### PFK is a link between PPARγ agonists-regulated FAO and UDP-GlcNAc/glycosylation axis

Beyond acetyl-CoA, fructose-6-phosphate is another cornerstone for UDP-GlcNAc biosynthesis, allowing HBP to be an important bypath of glycolysis [23]. Indeed, de novo synthesis of UDP-GlcNAc begins with the conversion of fructose-6-phosphate to glucosamine-6-phosphate through the rate-limiting enzyme GFPT, while fructose-6-phosphate is derived from glucose and enters glycolysis via the key regulatory enzyme PFK (Fig. 4a) [23, 24]. Therefore, the competition for fructose-6-phosphate by GFPT and PFK directs the glucose flux into HBP or glycolysis.

**Fig. 5:**
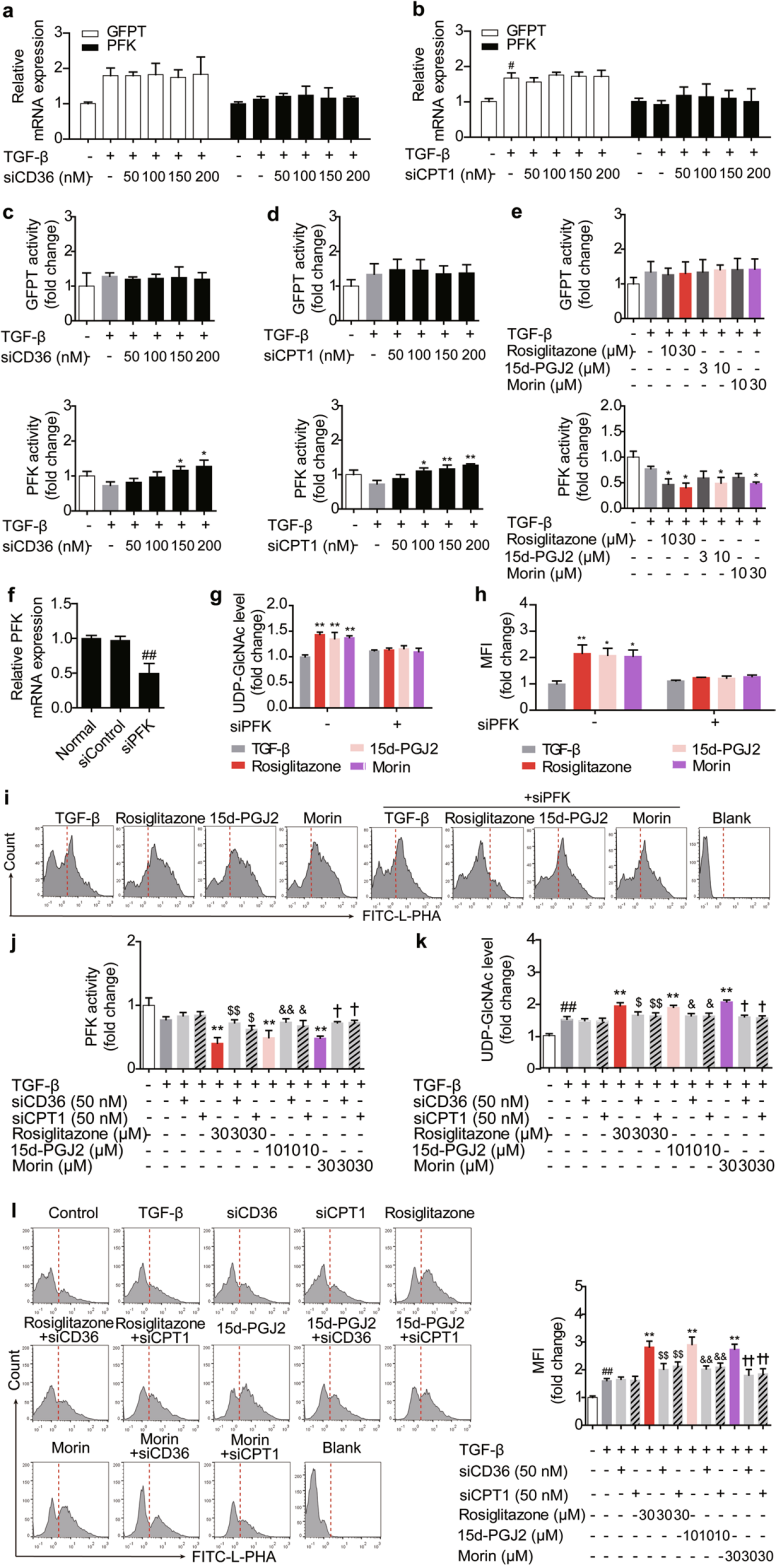
PFK participates in the effects of PPARγ agonists through associating FAO with UDP-GlcNAc/glycosylation axis. **a**–**d** The naïve CD4^+^ T cells were transfected with siCD36 (50, 100, 150, 200 nM) or siCPT1 (50, 100, 150, 200 nM), and treated with anti-CD3/CD28 in the presence or absence of TGF-β (5 ng/mL) for 48 h. The mRNA levels of GFPT and PFK were measured by Q-PCR (**a**, **b**), and the activity of PFK and GFPT was determined (**c**, **d**). **e** The naïve CD4^+^ T cells were prepared and treated with anti-CD3/CD28 in the presence or absence of TGF-β (5 ng/mL), rosiglitazone (10, 30 μM), 15d-PGJ2 (3, 10 μM) as well as morin (10, 30 μM) for 48 h. The activity of PFK and GFPT was determined. **f** The naïve CD4^+^ T cells were transfected with siPFK, and the mRNA level of PFK was measured by Q-PCR. **g**–**i** The naïve CD4^+^ T cells were transfected with siPFK, and treated with anti-CD3/CD28 in the presence or absence of TGF-β (5 ng/mL), rosiglitazone (30 μM), 15d-PGJ2 (10 μM) as well as morin (30 μM) for 48 h. The intracellular level of UDP-GlcNAc was determined (**g**), and the level of N-linked glycosylation was examined by flow cytometry using FITC-L-PHA (**h**, **i**). **j**–**l** The naïve CD4^+^ T cells were transfected with siCD36 or siCPT1, and treated with anti-CD3/CD28 in the presence or absence of TGF-β (5 ng/mL), rosiglitazone (30 μM), 15d-PGJ2 (10 μM) as well as morin (30 μM) for 48 h. The activity of PFK (**j**) and the intracellular level of UDP-GlcNAc (**k**) were determined. The level of N-linked glycosylation was examined by flow cytometry using FITC-L-PHA (**l**). Data were presented as the means ± S.E.M. of three independent experiments (n = 3). ^##^*P* < 0.01 versus control group; *P < 0.05, **P < 0.01 versus TGF-β group (model group); ^$^*P* < 0.05, ^$$^*P* < 0.01 versus rosiglitazone group; ^&^*P* < 0.05, ^&&^*P* < 0.01 versus 15d-PGJ2 group; ^†^*P* < 0.05, ^††^*P* < 0.01 versus morin group

To determine whether FAO exerted a regulatory effect on GFPT or PFK during Treg differentiation, the detection of mRNA expression based on Q-PCR assay was carried out. However, hardly any change in mRNA levels of GFPT or PFK could be observed in CD36- or CPT1-defficient cells (Fig. 5a, b). Research and theoretical developments have offered an alternative perspective, as the activity of PFK can be limited by citrate and ATP, two important products of tricarboxylic acid (TCA) cycle secondary to FAO [25–27]. It was revealed in Fig. 5c, d that the activity of PFK was enhanced during Treg differentiation when FAO was limited. On the contrary, the activity of PFK was dampened by all of three PPARγ agonists (Fig. 5e). In the case of GFPT, no obvious alteration in its activity caused by disturbing FAO or PPARγ agonists could be seen (Fig. 5c–e). PFK was further confirmed to be a link between FAO and UDP-GlcNAc/glycosylation axis in the action of PPARγ agonists, as PPARγ agonists could no longer up-regulate the level of UDP-GlcNAc and N-glycan branching when PFK was deficient (Fig. 5f–i) and the inhibitory effects of PPARγ agonists on PFK activity could be disrupted by siCD36 and siCPT1 (Fig. 5j). Moreover, by knocking down CD36 or CPT1, the effects of PPARγ agonists on UDP-GlcNAc level and N-glycan branching almost disappeared (Fig. 5k, l). Together, PPARγ agonists can favor glucose flux into HBP through limiting PFK activity as a result of the up-regulated FAO.

### PPARγ agonists enhance cell surface retention of TβRII and IL-2Rα through regulating N-linked glycosylation

**Fig. 6:**
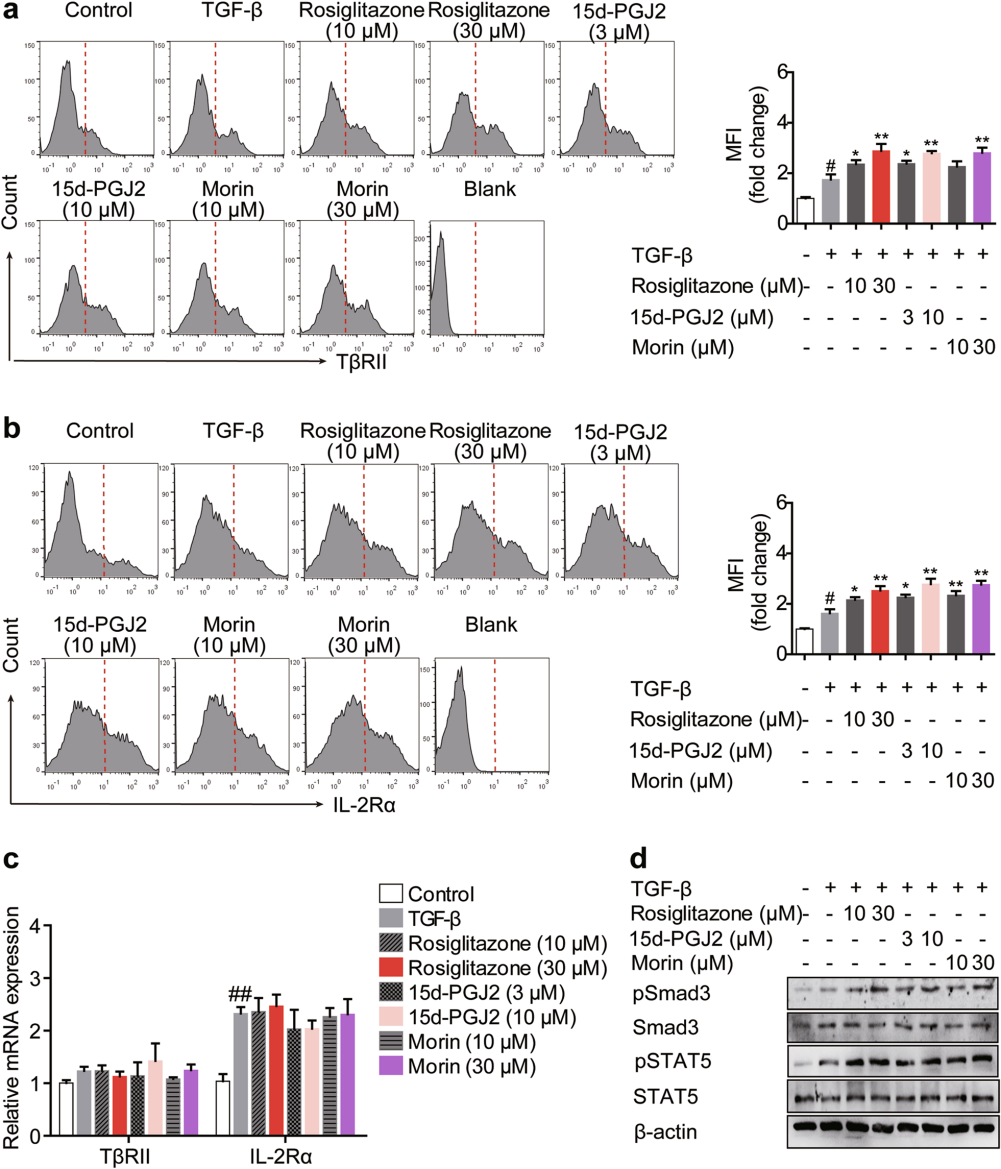
PPARγ agonists enhance cell surface retention of TβRII and IL-2Rα as well as downstream signaling. The naïve CD4^+^ T cells were prepared and treated with anti-CD3/CD28 in the presence or absence of TGF-β (5 ng/mL), rosiglitazone (10, 30 μM), 15d-PGJ2 (3, 10 μM) as well as morin (10, 30 μM) for 48 h. **a**, **b** The cell surface levels of TβRII (**a**) and IL-2Rα (CD25) (**b**) were analyzed by flow cytometry. **c** The mRNA expression levels of TβRII and IL-2Rα (CD25) were measured by Q-PCR. **d** The protein levels of pSmad3, Smad3, pSTAT5 and STAT5 were detected by western blotting. Data were presented as the means ± S.E.M. of three independent experiments (n = 3). ^#^*P* < 0.05, ^##^*P* < 0.01 versus control group; *P < 0.05, **P < 0.01 versus TGF-β group (model group)

**Fig. 7:**
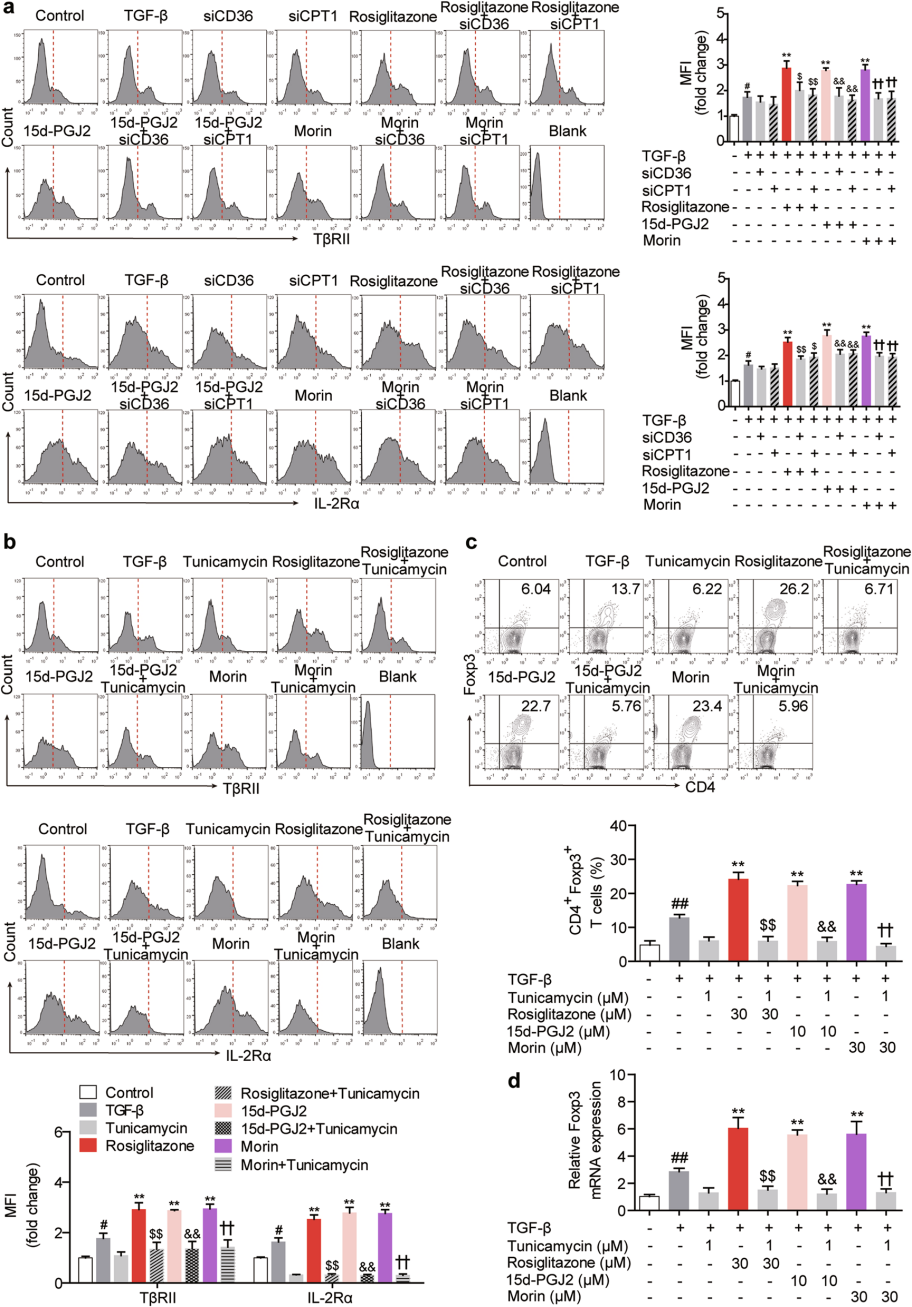
Surface retention of TβRII/IL-2Rα resulting from glycosylation links PPARγ agonists-boosted FAO and Treg responses. **a** The naïve CD4^+^ T cells were transfected with siCD36 or siCPT1, and treated with anti-CD3/CD28 in the presence or absence of TGF-β (5 ng/mL), rosiglitazone (30 μM), 15d-PGJ2 (10 μM) as well as morin (30 μM) for 48 h. The cell surface levels of TβRII and IL-2Rα (CD25) were analyzed by flow cytometry. **b**–**d** The naïve CD4^+^ T cells were prepared and treated with anti-CD3/CD28 in the presence or absence of TGF-β (5 ng/mL), tunicamycin (1 μM), rosiglitazone (30 μM), 15d-PGJ2 (10 μM) as well as morin (30 μM). After 48 h, the cell surface levels of TβRII and IL-2Rα (CD25) (**b**) were analyzed by flow cytometry. After 72 h, the frequency of CD4^+^Foxp3^+^ T cells was examined by flow cytometry (**c**) and the mRNA expression level of Foxp3 was measured by Q-PCR (**d**). Data were presented as the means ± S.E.M. of three independent experiments (n = 3). ^#^*P* < 0.05, ^##^*P* < 0.01 versus control group; **P < 0.01 versus TGF-β group (model group); ^$^*P* < 0.05, ^$$^*P* < 0.01 versus rosiglitazone group; ^&&^*P* < 0.01 versus 15d-PGJ2 group; ^††^*P* < 0.01 versus morin group

As N-glycan branching facilitates cell surface retention/localization of multiple receptors [28], the effects of PPARγ agonists on classical Treg-favoring TGF-β/Smads and IL-2/STAT5 pathways, which are reliant on membrane TβRII and IL-2Rα respectively, were investigated. It was showed by flow cytometry that rosiglitazone (10, 30 μM), 15d-PGJ2 (3, 10 μM) and morin (10, 30 μM) obviously elevated the cell surface levels of TβRII and IL-2Rα in differentiating Treg cells, although the expressions of the two receptors were not altered, implying a promoting effect of PPARγ agonists on their cell surface retention/localization (Fig. 6a–c). Expectedly, the phosphorylation of Smad3 and STAT5 was up-regulated by rosiglitazone (10, 30 μM), 15d-PGJ2 (3, 10 μM) and morin (10, 30 μM), which represented the activation of the downstream signals of TβRII and IL-2Rα (Fig. 6d). However, when the cells were treated with siCD36, siCPT1 or tunicamycin (an inhibitor of N-glycan branching; 1 μM), the effects of PPARγ agonists on cell surface retention of TβRII and IL-2Rα were abolished (Fig. 7a, b). Beyond N-linked glycosylation, O-GlcNAcylation is another kind of glycosylation. To confirm the specific effect of N-linked glycosylation on TβRII and IL-2Rα in the action of PPARγ agonists, OSMI-1, an O-GlcNAc transferase (OGT) inhibitor which specially restricts O-GlcNAcylation, was additionally used. Different from tunicamycin, only a weak limiting effect of OSMI-1 could be seen on the membrane abundance of TβRII and IL-2Rα up-regulated by PPARγ agonists (Additional file 2: Fig. S2). What’s more, the frequency of Treg cells and the mRNA expression of Foxp3 up-regulated by PPARγ agonists were restored by tunicamycin (Fig. 7c, d). These findings indicate that, by regulating N-linked glycosylation, PPARγ agonists enhance the cell surface retention of TβRII and IL-2Rα, thus leading to the activation of TGF-β/Smads and IL-2/STAT5 signaling and the promotion of subsequent Treg differentiation.

### The increased surface abundance of CD36 mainly results from the up-regulation of its overall expression by PPARγ agonists

**Fig. 8:**
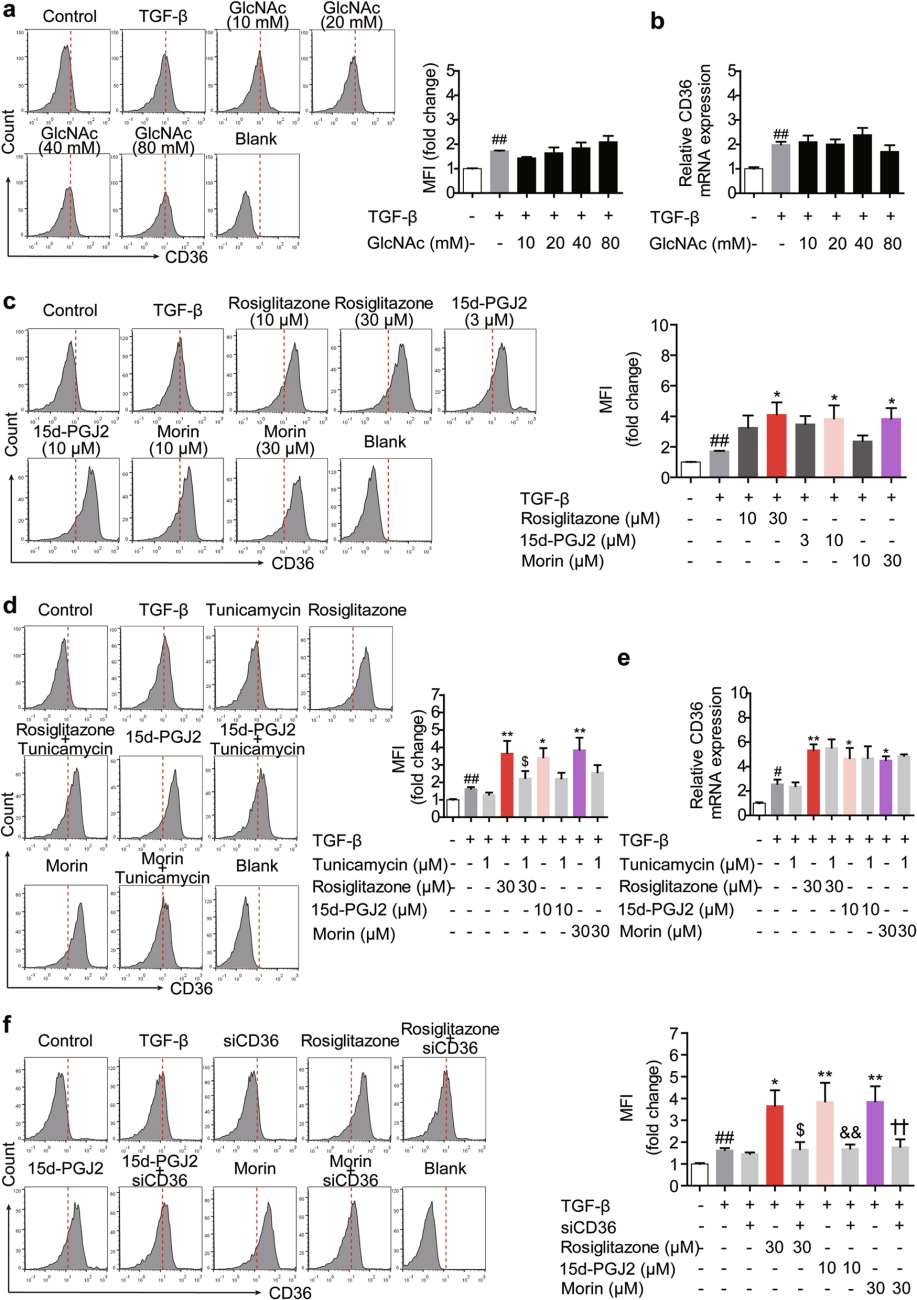
The increased cell surface level of CD36 is mainly attributed to the regulation of its overall expression by PPARγ agonists. **a**, **b** The naïve CD4^+^ T cells were prepared and treated with anti-CD3/CD28 in the presence or absence of GlcNAc (10, 20, 40, 80 mM) for 48 h. The cell surface level of CD36 were analyzed by flow cytometry (**a**), and the mRNA expression level of CD36 was measured by Q-PCR (**b**). **c** The naïve CD4^+^ T cells were prepared and treated with anti-CD3/CD28 in the presence or absence of TGF-β (5 ng/mL), rosiglitazone (10, 30 μM), 15d-PGJ2 (3, 10 μM) as well as morin (10, 30 μM) for 48 h. The cell surface level of CD36 was analyzed by flow cytometry. **d**, **e** The naïve CD4^+^ T cells were prepared and treated with anti-CD3/CD28 in the presence or absence of TGF-β (5 ng/mL), tunicamycin (1 μM), rosiglitazone (30 μM), 15d-PGJ2 (10 μM) as well as morin (30 μM) for 48 h. The cell surface level of CD36 was analyzed by flow cytometry (**d**), and the mRNA expression level of CD36 was measured by Q-PCR (**e**). **f** The naïve CD4^+^ T cells were transfected with siCD36, and treated with anti-CD3/CD28 in the presence or absence of TGF-β (5 ng/mL), rosiglitazone (30 μM), 15d-PGJ2 (10 μM) as well as morin (30 μM) for 48 h. The cell surface level of CD36 was analyzed by flow cytometry. Data were presented as the means ± S.E.M. of three independent experiments (n = 3). ^#^*P* < 0.05, ^##^*P* < 0.01 versus control group; *P < 0.05, **P < 0.01 versus TGF-β group (model group); ^$^*P* < 0.05, ^$$^*P* < 0.01 versus rosiglitazone group; ^&^*P* < 0.05, ^&&^*P* < 0.01 versus 15d-PGJ2 group; ^†^*P* < 0.05, ^††^*P* < 0.01 versus morin group

Considering that CD36 is a fatty acid transporter whose location may be under the control of N-linked glycosylation during Treg differentiation as well, the cell surface retention/localization of CD36 was additionally examined, and the UDP-GlcNAc was generated directly by exploiting the HBP where GlcNAc was used in order to bypass the effect of GFPT competition for fructose-6-phosphate. However, only a slight increasing effect was shown on the surface abundance of CD36 in cells supplemented with GlcNAc, while the expression of CD36 was not affected (Fig. 8a, b). Therefore, although rosiglitazone (10, 30 μM), 15d-PGJ2 (3, 10 μM) and morin (10, 30 μM) increased the membrane level of CD36 under Treg-skewing conditions, and tunicamycin could constrain the effect to some extent without altering CD36 expression (Fig. 8c–e), glycosylation-related localization was not the deciding factor in the action of PPARγ agonists. Intriguingly, unlike GlcNAc, PPARγ agonists caused the up-regulation of overall expression of CD36, which might be the possible reason giving rise to the increased the membrane level of CD36. The hypothesis was confirmed by treatment with siCD36, as disturbing CD36 expression markedly alleviated the effect of PPARγ agonists on its surface abundance (Fig. 8f). These data suggest PPARγ agonists regulate the membrane location of TβRII, IL-2Rα and CD36 by different means.

### The regulation of CD36/CPT1 and Treg responses by PPARγ agonists is dependent on PPARγ

**Fig. 9:**
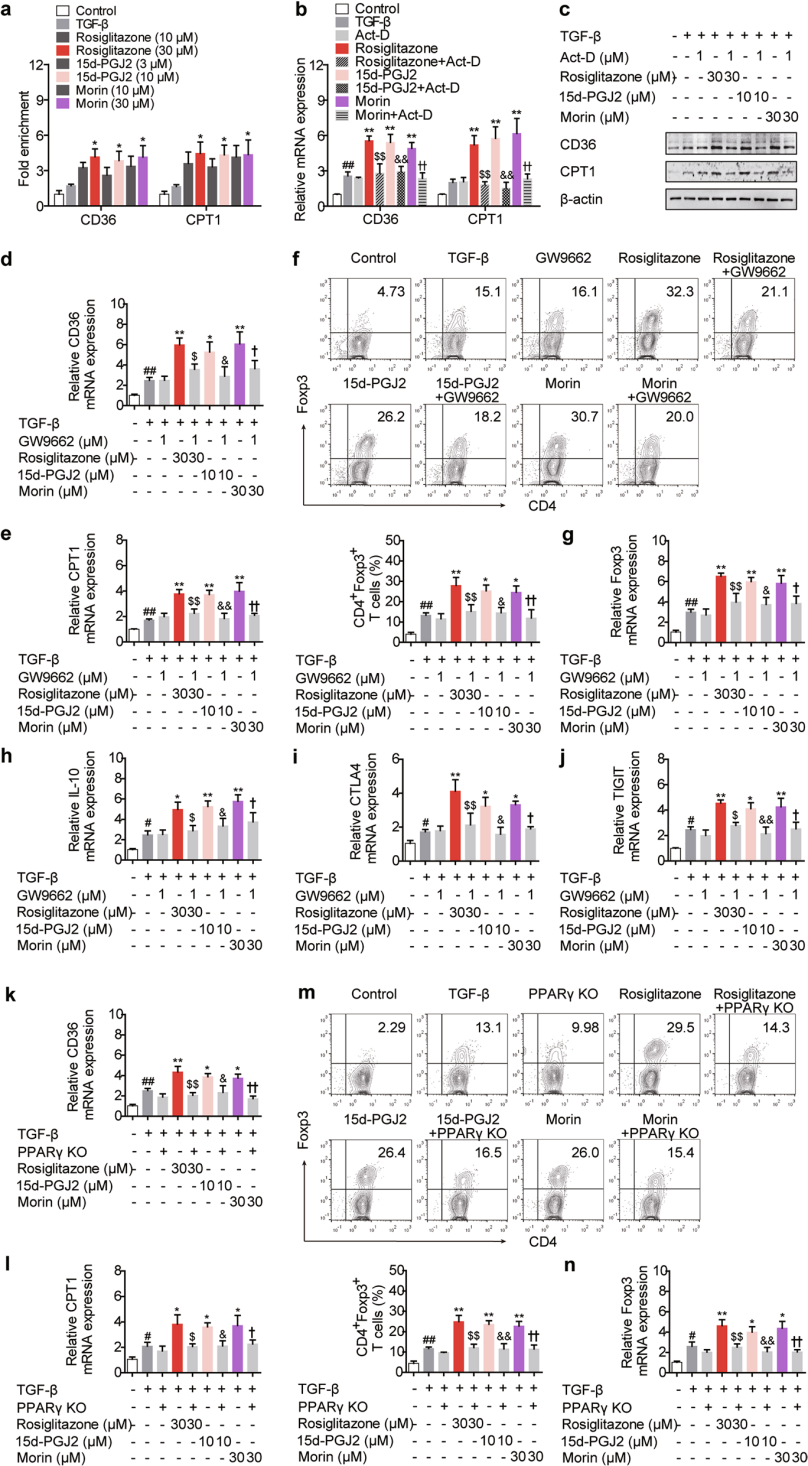
The regulation of PPARγ agonists on CD36/CPT1-mediated FAO and Treg responses exerts a PPARγ-dependent feature. **a** The naïve CD4^+^ T cells were prepared and treated with anti-CD3/CD28 in the presence or absence of TGF-β (5 ng/mL), rosiglitazone (10, 30 μM), 15d-PGJ2 (3, 10 μM) as well as morin (10, 30 μM) for 48 h. The binding of PPARγ to the promoter of CD36/CPT1 was detected by ChIP assay. **b**, **c** The naïve CD4^+^ T cells were prepared and treated with anti-CD3/CD28 in the presence or absence of TGF-β (5 ng/mL), actinomycin D (Act-D; 1 μM), rosiglitazone (30 μM), 15d-PGJ2 (10 μM) as well as morin (30 μM) for 48 h. The mRNA expression levels of CD36 and CPT1 were measured by Q-PCR (**b**), and the protein levels of CD36 and CPT1 were detected by western blotting (**c**). **d**–**j** The naïve CD4^+^ T cells were prepared and treated with anti-CD3/CD28 in the presence or absence of TGF-β (5 ng/mL), GW9662 (1 μM), rosiglitazone (30 μM), 15d-PGJ2 (10 μM) as well as morin (30 μM). After 48 h, the mRNA expression levels of CD36 (**d**) and CPT1 (**e**) were measured by Q-PCR. After 72 h, the frequency of CD4^+^Foxp3^+^ T cells was examined by flow cytometry (**f**), and the mRNA expression levels of Foxp3 (**g**), IL-10 (**h**), CTLA4 (**i**) and TIGIT (**j**) were measured by Q-PCR. **k**–**n** The naïve CD4^+^ T cells were transfected with PPARγ KO plasmid and treated with anti-CD3/CD28 in the presence or absence of TGF-β (5 ng/mL), rosiglitazone (30 μM), 15d-PGJ2 (10 μM) as well as morin (30 μM). After 48 h, the mRNA expression levels of CD36 (**k**) and CPT1 (**l**) were measured by Q-PCR. After 72 h, the frequency of CD4^+^Foxp3^+^ T cells was examined by flow cytometry (**m**), and the mRNA expression level of Foxp3 (**n**) was measured by Q-PCR. Data were presented as the means ± S.E.M. of three independent experiments (n = 3). ^#^*P* < 0.05, ^##^*P* < 0.01 versus control group; *P < 0.05, **P < 0.01 versus TGF-β group (model group); ^$^*P* < 0.05, ^$$^*P* < 0.01 versus rosiglitazone group; ^&^*P* < 0.05, ^&&^*P* < 0.01 versus 15d-PGJ2 group; ^†^*P* < 0.05, ^††^*P* < 0.01 versus morin group

Aside from PPARγ, rosiglitazone, 15d-PGJ2 and morin can function through other targets [29–31]. Of note, both CD36 and CPT1 have been reported to be PPARγ target genes [32, 33]. Since rosiglitazone (30 μM), 15d-PGJ2 (10 μM) and morin (30 μM) enhanced the binding of PPARγ to the promoter of CD36/CPT1 gene, and the RNA synthesis inhibitor Act-D restrained the elevated expression of CD36/CPT1 caused by them (Fig. 9a–c), it could be reached that these PPARγ agonists did activate PPARγ, thereby boosting the transcription of CD36 and CPT1. Furthermore, the involvement of PPARγ in rosiglitazone-, 15d-PGJ2- and morin-regulated mRNA expression of CD36/CPT1 was confirmed by combination treatment with the PPARγ antagonist GW9662 (1 μM), and the CRISPR/Cas9 KO plasmid for knocking out PPARγ was used in a more comprehensive experiment, where similar results were obtained as indicated by Fig. 9d, e, k, l. In addition, Fig. 9f–j, m, n revealed that, by interfering with PPARγ, the promoting effects of rosiglitazone (30 μM), 15d-PGJ2 (10 μM) and morin (30 μM) on the frequency of CD4^+^Foxp3^+^ T cells as well as the mRNA expressions of Foxp3, IL-10, CTLA4 and TIGIT almost disappeared. These results indicate the exact role PPARγ plays in rosiglitazone-, 15d-PGJ2- and morin-regulated CD36/CPT1 expression as well as Treg responses.

## Discussion

Treg cells are the major gatekeepers of immune system for the maintenance of immune homeostasis. Upon T cell receptor (TCR) ligation and co-stimulation, naïve CD4^+^ T cells are activated and develop into Treg cells when stimulated by TGF-β, or along with IL-2. Through the secretion of inhibitory cytokines such as IL-10 or cell-to-cell crosstalk mechanisms which are contact-dependent and include the expressions of inhibitory molecules such as CTLA4, TIGIT, etc., Treg cells are able to suppress the activation and proliferation of effector cells [1, 34]. Although the role of PPARγ, a ligand-dependent transcription factor of the nuclear receptor superfamily that attracts intensive research endeavors, in controlling Treg differentiation and function has been unveiled, more experiments are still needed to garner an understanding of the underlying mechanisms. In this study, three PPARγ agonists were used for an attempt to solve this problem. Among them, rosiglitazone is a synthetic compound belonging to thiazolidinediones, which have long been considered as the classical PPARγ agonists [35]. 15d-PGJ2, the most widely studied cyclopentenone prostaglandin, is the first identified endogenous ligand of PPARγ [36]. Morin, a natural flavonoid exists in *Morus alba* L. (Moraceae) and *Otostegia persica* (Lamiaceae), has recently been recognized as a PPARγ agonist in our laboratory by carrying out the competitive binding assay together with the luciferase reporter gene assay [37]. As expected, the results revealed the boosting effects of rosiglitazone, 15d-PGJ2 and morin on Treg responses, which were attributed to the up-regulated transcription of Foxp3, allowing them to be the ideal tool drugs for further research.

In mechanistic studies, attention was focused on FAO, which might be a potential link between PPARγ and Treg responses, as the β-oxidation of exogenous fatty acid fuels Treg cells, while PPARγ is a master regulator of energy metabolism including FAO [10–17]. The process of exogenous FAO is complex [21, 38]. Under the action of fatty acid transporter CD36 and so on, exogenous fatty acids enter the cytoplasm and are activated to fatty acyl-CoA before shuttling into mitochondria. Subsequently, fatty acyl-CoA is converted to fatty acyl-carnitine by CPT1 on the outer mitochondrial membrane, permitting the entry into mitochondria where fatty acyl-carnitine is converted back to fatty acyl-CoA. The majority of this fatty acyl-CoA then enters the β-oxidation cycle, producing acetyl-CoA fragments until the fatty acyl-CoA is completely degraded. Our previous research has revealed that a natural PPARγ agonist, madecassic acid, can up-regulate the level of CD36 as well as the frequency of Treg cells in dextran sulfate sodium (DSS)-induced colitis mice [39]. Another published paper has reported that, by preventing the degradation of PPARγ, the expression of CD36 can be enhanced, while the Th17/Treg balance can be restored in mice with colitis, implying the involvement of fatty acid oxidation (FAO) in the effects of PPARγ on Treg responses in vivo [40]. In this study, it was showed that PPARγ agonists facilitated the transport of fatty acids, as rosiglitazone, 15d-PGJ2 and morin had a promoting effect on the expressions of CD36 and CPT1, which imported fatty acids into the cell and mitochondria respectively, thereby enhancing FAO and Treg differentiation.

Recently, reports have indicated that the biosynthesis of UDP-GlcNAc and subsequent N-linked glycosylation play an essential role in determining the Treg phenotype. Abolishing N-glycan branching via Mgat1/Mgat5 deficiency or using branching inhibitor kifunensine eliminates Treg differentiation induced by TGF-β, while raising branching with GlcNAc has the opposite effect [24]. It is worth noting that FAO may have a link with UDP-GlcNAc biosynthesis in two ways. On one hand, by providing the acetyl group, the resulting acetyl-CoA from FAO is directly involved in the formation of *N*-acetylglucosamine-6-phosphate (GlcNAc-6P), an important intermediate in UDP-GlcNAc biosynthesis [41]. On the other hand, FAO may participate in UDP-GlcNAc biosynthesis through an indirect mechanism. In detail, acetyl-CoA derived from FAO can enter the TCA cycle for further oxidization, leading to the accumulation of two PFK allosteric inhibitors, citrate and ATP, and thus has a role in regulating the availability of fructose-6-phosphate for UDP-GlcNAc biosynthesis by modulating the activity of PFK [24–27]. That means the heightened FAO caused by PPARγ agonists is possible to enhance UDP-GlcNAc biosynthesis and subsequent N-linked glycosylation during Treg differentiation. The results in this study confirmed the conjecture, and the connection between PPARγ agonists-enhanced FAO and UDP-GlcNAc/glycosylation axis under Treg-skewing conditions was actually revealed.

In mammalian cells, most receptors and transporters at cell surface are glycoproteins modified with N-glycans in the endoplasmic reticulum and Golgi apparatus. Branched N-glycans can bind galectins and form the lattice at cell surface so as to facilitate the membrane retention/localization of these receptors and transporters, thereby providing adaptive control over cell growth and differentiation [28, 42]. In the case of T cells, sufficient surface abundance of TβRII and IL-2Rα permits the cellular sensitivity to TGF-β and IL-2, two Treg-skewing cytokines in the extracellular environment [43–45]. Upon ligand-receptor binding, the constitutively active TβRII is able to phosphorylate the intracellular kinase domain of TβRI, which then propagates the signal through phosphorylation of the downstream Smad proteins, while the binding of IL-2 to IL-2Rα causes the activation of JAK-STAT pathway, particularly JAK1 and JAK3, which in turn phosphorylate STAT5 [43, 45]. Finally, both the activation of TGF-β/Smads and IL-2/STAT5 signaling pathways can result in the assembling of transcription factors on the Foxp3 locus and consequent transcription, thus leading to the induction of Treg generation and function [44, 46]. Therefore, we explored how PPARγ agonists-enhanced UDP-GlcNAc/glycosylation axis participated in Treg responses from the perspective of regulating these two pathways, and found that PPARγ agonists could increase the cell surface abundance of TβRII and IL-2Rα via N-linked glycosylation, which promoted the subsequent signaling and Treg responses.

Beyond TβRII and IL-2Rα, fatty acid transporter CD36 is another protein of which the surface abundance is essential for its function [47]. However, although glycosylation of CD36 is necessary for membrane retention/localization, it seems to be a redundancy that partially glycosylated CD36 mutants are still properly distributed to the cell surface [48]. In addition, different from TβRII and IL-2Rα, which have low density of N-glycans (N-linked glycosylation sites: n ≤ 4), CD36 has 10 N-linked glycosylation sites located in the extracellular segment [48–50]. At low level of branching, glycoprotein with higher density of N-glycans is capable of generating significant avidity for galectins, while high level of branching is required by glycoprotein with low density of N-glycans to incorporate into the galectin lattice and maintenance at the cell surface [49]. Thus, distinctive responses to the increasing UDP-GlcNAc concentration may exhibit as have been noted previously [51], and it is possible that upon TCR and TGF-β stimulation, the intracellular level of UDP-GlcNAc is enough to facilitate the membrane retention/localization of CD36, resulting in the slight change of its surface abundance caused by further supplementing GlcNAc. Moreover, it was reached in this study that PPARγ agonists could break down the barriers by expanding the CD36 pool (boosting the expression of CD36), leading to the up-regulated cell surface level of CD36.

Of note, although rosiglitazone, 15d-PGJ2 and morin are PPARγ agonists, several lines of evidence suggest that they can function through other targets instead of PPARγ. For example, rosiglitazone dampens the expression of pigment epithelium-derived factor (PEDF), a driver of insulin resistance, in hepatocytes and adipocytes by activating AMP-activated protein kinase (AMPK) rather than PPARγ [29]. 15d-PGJ2 functions as an inhibitor of NF-κB to dampen the NLR family leucine-rich repeat protein (NLRP)1 and NLRP3 inflammasomes independent of PPARγ [30]. Morin can suppress cell chemotaxis and nitric oxide production in RAW264.7 cells by inhibiting the tautomerase activity of macrophage migration inhibitory factor (MIF) [31]. In this study, we excluded this possibility, as rosiglitazone, 15d-PGJ2 and morin could boost the transcription of CD36 and CPT1, two target genes of PPARγ [32, 33], and the effects of them on CD36 and CPT1 and subsequent Treg responses were rescued by using the PPARγ antagonist GW9662 or knocking out the PPARγ.

## Conclusions

In summary, the activation of PPARγ boosts Treg responses through up-regulating FAO and subsequent N-glycan branching. The precise mechanisms can be summarized as follows: boosting the expressions of CD36/CPT1, up-regulating FAO, enhancing UDP-GlcNAc biosynthesis, promoting the N-linked glycosylation of TβRII/IL-2Rα as well as the downstream signaling and thus increasing the transcription of Foxp3.

## Supplementary Information

**Additional file 1: Fig. S1.** Effects of PPARγ agonists on cell proliferation and apoptosis during Treg differentiation. The naïve CD4^+^ T cells were prepared and treated with anti-CD3/CD28 in the presence or absence of TGF-β (5 ng/mL), rosiglitazone (10, 30 μM), 15d-PGJ2 (3, 10 μM) as well as morin (10, 30 μM) for 72 h. **a** Cell proliferation was determined by Edu staining (scale bars: 50 μm). **b **Cell apoptosis was detected by Annexin V-FITC/PI staining and flow cytometry. Data were presented as the means ± S.E.M. of three independent experiments (n = 3).

**Additional file 2: Fig. S2.** Effects of OSMI-1 on PPARγ agonists-increased surface abundance of TβRII/IL-2Rα. The naïve CD4^+^ T cells were prepared and treated with anti-CD3/CD28 in the presence or absence of TGF-β (5 ng/mL), OSMI-1 (20 μM) rosiglitazone (30 μM), 15d-PGJ2 (10 μM) as well as morin (30 μM) for 48 h. **a**, **b** The cell surface levels of TβRII (**a**) and IL-2Rα (CD25) (**b**) were analyzed by flow cytometry. Data were presented as the means ± S.E.M. of three independent experiments (n = 3). ^#^*P* < 0.05 vs. Control group; *P < 0.05, **P < 0.01 vs. TGF-β group (model group).

## Data Availability

All data generated or analyzed during this study are included in this published article.

## Abbreviations

Acetyl-CoA: Acetyl-coenzyme A
FAO: Fatty acid oxidation
Foxp3: Forkhead box protein 3
GFPT: Glutamine-fructose-6-phosphate transaminase
GlcNAc: *N*-acetylglucosamine
HBP: Hexosamine biosynthetic pathway
PFK: Phosphofructokinase
PPARγ: Peroxisome proliferator-activated receptor gamma
Treg: Regulatory T cells
UDP-GlcNAc: Uridine diphosphate-*N*-acetylglucosamine
