# Supplementary figures and images for: The activation of PPARγ enhances Treg responses through up-regulating CD36/CPT1-mediated fatty acid oxidation and subsequent N-glycan branching of TβRII/IL-2Rα
Source: Cell Commun Signal. 2022 Apr 7;20:48. doi: 10.1186/s12964-022-00849-9 (PMC8991706; doi:10.1186/s12964-022-00849-9)

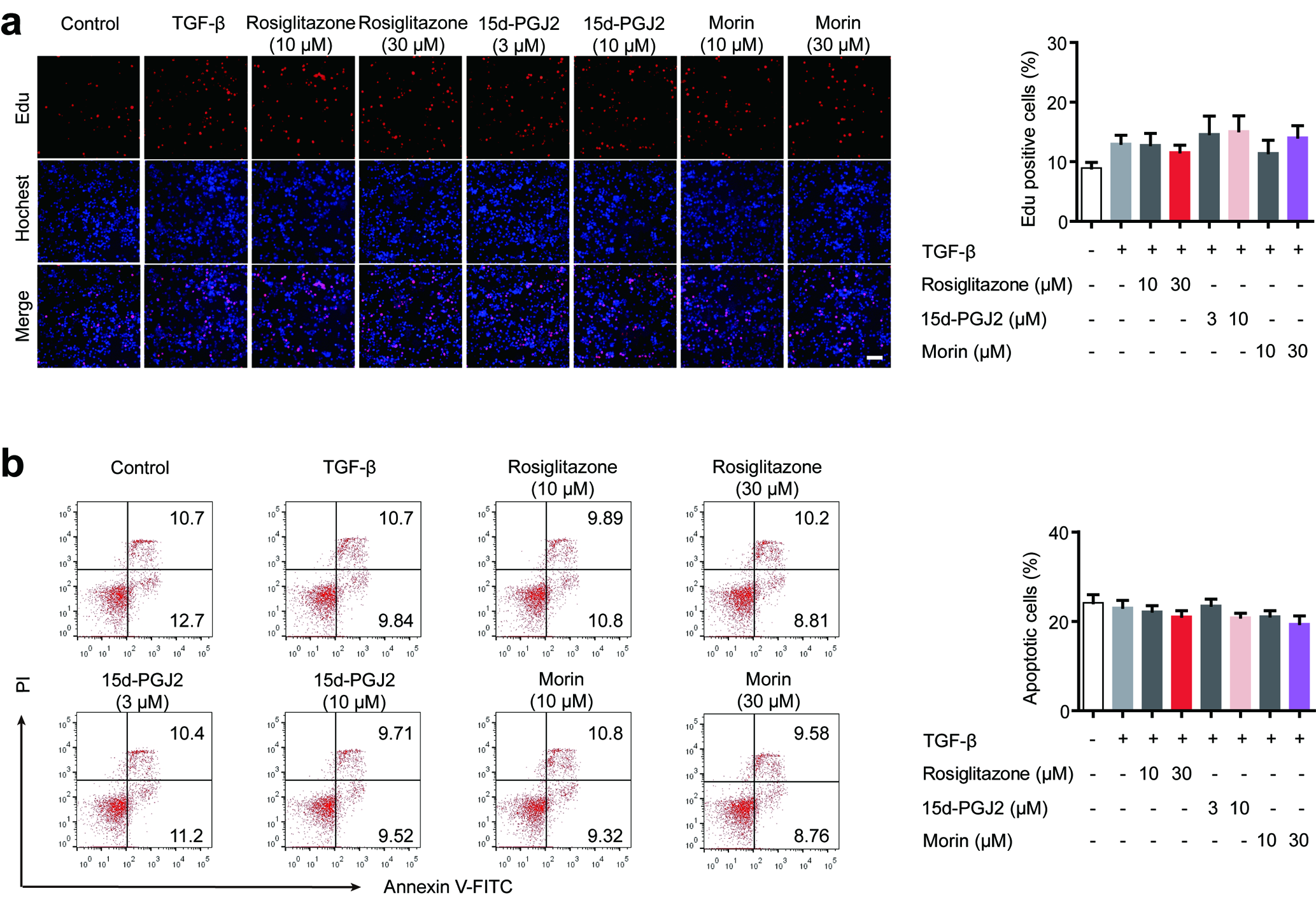

Supplement: Supplementary file 2 — Additional file 1: Fig. S1. Effects of PPARγ agonists on cell proliferation and apoptosis during Treg differentiation. The naïve CD4+ T cells were prepared and treated with anti-CD3/CD28 in the presence or absence of TGF-β (5 ng/mL), rosiglitazone (10, 30 μM), 15d-PGJ2 (3, 10 μM) as well as morin (10, 30 μM) for 72 h. a Cell proliferation was determined by Edu staining (scale bars: 50 μm). b Cell apoptosis was detected by Annexin V-FITC/PI staining and flow cytometry. Data were presented as the means ± S.E.M. of three independent experiments (n = 3). [file 12964_2022_849_MOESM2_ESM.tif]

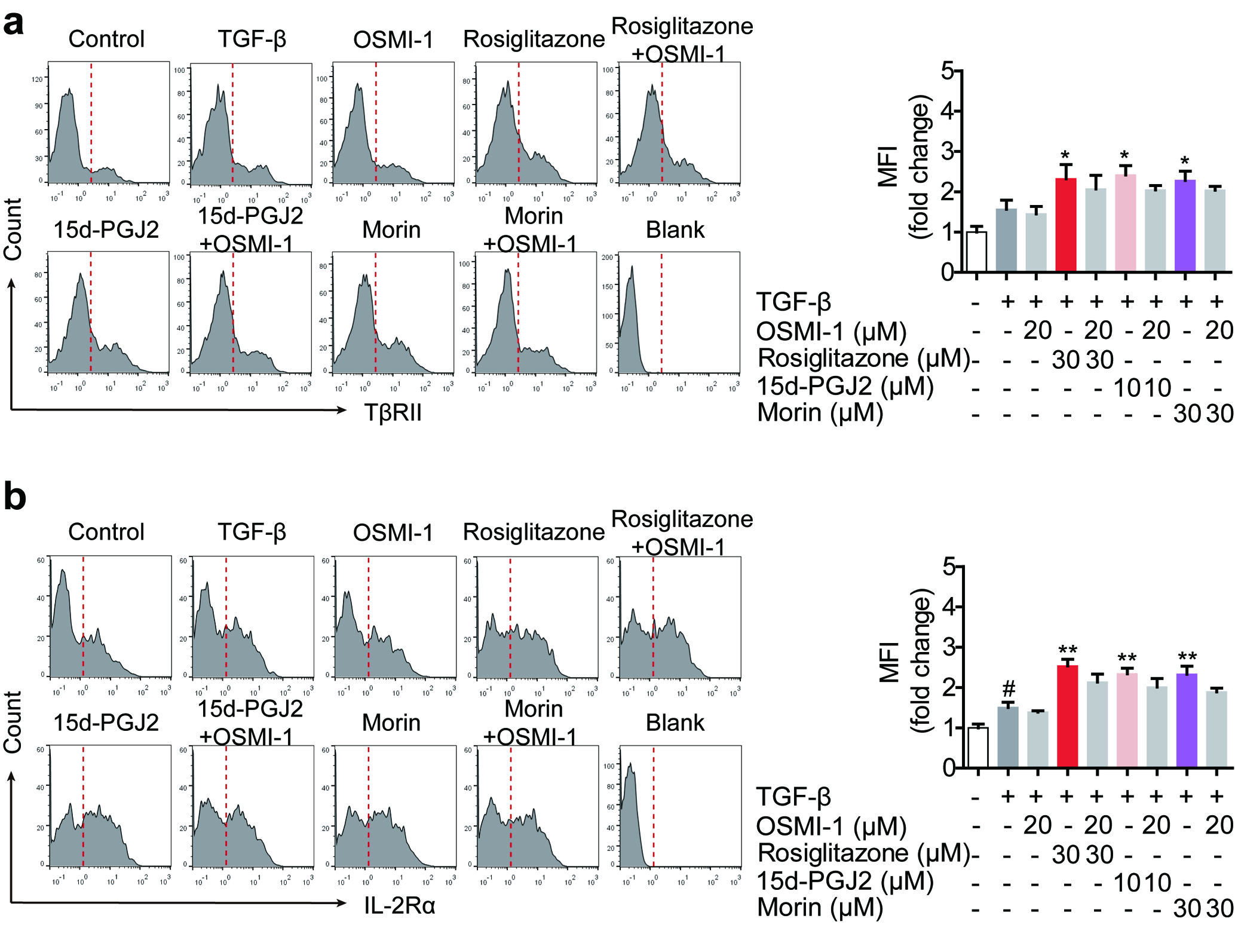

Supplement: Supplementary file 3 — Additional file 2: Fig. S2. Effects of OSMI-1 on PPARγ agonists-increased surface abundance of TβRII/IL-2Rα. The naïve CD4+ T cells were prepared and treated with anti-CD3/CD28 in the presence or absence of TGF-β (5 ng/mL), OSMI-1 (20 μM) rosiglitazone (30 μM), 15d-PGJ2 (10 μM) as well as morin (30 μM) for 48 h. a, b The cell surface levels of TβRII (a) and IL-2Rα (CD25) (b) were analyzed by flow cytometry. Data were presented as the means ± S.E.M. of three independent experiments (n = 3). #P < 0.05 vs. Control group; *P < 0.05, **P < 0.01 vs. TGF-β group (model group). [file 12964_2022_849_MOESM3_ESM.tif]
